# Ivermectin decreases inflammation and imiquimod–induced psoriasis-like skin lesions in rat via targeting TLR4/p65 NF-κB

**DOI:** 10.22038/ijbms.2025.83254.18008

**Published:** 2025

**Authors:** Tayebeh Noori, Antoni Sureda, Samira Shirooie

**Affiliations:** 1 Pharmaceutical Sciences Research Center, Health Institute, Kermanshah University of Medical Sciences, Kermanshah, Iran; 2 Research Group on Community Nutrition and Oxidative Stress (NUCOX) and Health Research Institute of Balearic Islands (IdISBa), University of Balearic Islands-IUNICS, Palma de Mallorca E-07122, Balearic Islands, Spain; 3 CIBER Fisiopatología de la Obesidad y Nutrición (CIBEROBN), Instituto de Salud Carlos III (ISCIII), 28029 Madrid, Spain

**Keywords:** Cytokine, Imiquimod, Psoriasis, Rat, TLR4, Topical ivermectin

## Abstract

**Objective(s):**

Psoriasis is a chronic skin disease that usually manifests as white and silver spots on the skin. Because of its anti-inflammatory properties, we investigated the effects of ivermectin (IVM) on imiquimod (IMQ)-induced psoriasis in rats.

**Materials and Methods:**

Fifteen rats were assigned to 3 different groups (n=5 per group): the control group received normal water and food; the psoriasis group, in which psoriasis was induced by topical application of IMQ (1 mg per rat), and treatment group where rats were treated daily with topical IVM-gel (1%) from day 3 to 7. The Psoriasis Area Severity Index (PASI) Score for the entire treatment period was used to assess erythema, silver scale, and skin thickness on the dorsal region of rats, and the spleen-to-body weight index on day 7 was examined. Moreover, histological assessment of skin tissues was performed using fluorescence immunostaining and hematoxylin-eosin (H&E) staining.

**Results:**

The severity of lesions in the ivermectin group was reduced compared to the IMQ group, with a significant decrease in the average PASI scores. The results of fluorescence immunostaining showed that topical administration of IVM-gel reduced inflammation by decreasing Toll-like receptor 4 (TLR4) levels and p65 nuclear factor kappa-B (NF-κB). Furthermore, findings from H&E staining revealed that IVM-gel decreased dermal fibrosis, epidermal thickness, and infiltration of inflammatory cells caused by IMQ.

**Conclusion:**

Based on the obtained results, it can be concluded that IVM-gel can effectively reduce psoriasis lesions due to its therapeutic properties, such as anti-inflammatory effects via targeting TLR4/p65 NF-κB.

## Introduction

Psoriasis is a non-contagious inflammatory skin condition with an unknown cause that leads to itching and abnormal epidermal growth (1, 2). Factors triggering psoriasis include genetics, streptococcal infections, skin damage, stress, smoking, alcohol consumption, obesity, and certain drugs such as propranolol, lithium, indomethacin, and antimalarial drugs (3, 4). This condition affects nearly 2–3% of the world’s population (5) and is characterized by symptoms such as dryness, itching, burning, red patches with silver scales, and joint swelling and stiffness (6). In addition, it leads to immune system dysfunction (7). Cytokines like IL-17A, IL-22, IL-23, and tumor necrosis factor-a (TNF-α) play a role in psoriasis pathogenesis (8). Malondialdehyde (MDA), as indicator of lipid peroxidation and oxidative stress, is an effective marker of several diseases, including psoriasis (9). Tanhapour and colleagues reported that psoriatic patients have higher plasma MDA concentrations than healthy individuals (10). 

Toll-like receptors (TLRs) are a category of membrane pattern recognition receptors (PRRs) that initiate signals in response to various pathogen-associated molecular patterns (PAMPs) (11). TLRs are present in all types of immune cells, including macrophages, monocytes, dendritic cells, neutrophils, and basophils, and non-immune cells such as epithelial and endothelial cells. In addition, TLRs are also present in the brain (12, 13). Evidence suggests that TLR4 is involved in the immune response in the psoriasis pathogenesis (14). Psoriasis inhibits TLR4 function on dendritic cells, leading to dendritic cell dysfunction, release of anti-inflammatory cytokines, and suppression of hypersensitivity reactions and inflammation (15). It has been shown that targeting and inhibiting TLR4 effectively prevents the incidence of auto-inflammatory symptoms in a mouse model of IL-36 receptor antagonist deficiency-induced psoriasis (16). Nuclear factor kappa B (NF-κB) is a critical regulator of pro-inflammatory gene expression, inducing the expression of cytokines such as TNF-a, IL-1b, IL-6, and IL-8 (17). In psoriasis, keratinocyte differentiation and proliferation can be regulated and modulated by many cytokine transcription factors and inflammatory mediators released from chronic inflammatory cells that accompany these lesions (18, 19). Since NF-κB regulates cytokine gene expression, inhibiting proliferation and inflammatory responses of keratinocytes through the inactivation of the NF-κB signaling pathway may represent a novel psoriasis treatment (20-22). 

Imiquimod (IMQ) is a modifier of safety response that acts as a TLR-7/8 agonist, and the topical application in mice induces psoriasis-like dermatitis (23, 24). IMQ is also used to treat skin conditions like keratosis and certain types of skin cancer, such as Superficial Basal Cell Carcinoma (25, 26). It is a common model of induction of psoriasis (27) as it activates the production of downstream factors like IL-6, IL-23, IL-1β, and TNF-α by binding to TLR-7 and stimulating epidermal plasma-like dendritic cells and macrophages (28). It has been shown that mice treated with IMQ exhibit epidermal alterations (parakeratosis, acanthosis), skin erythema, scaling, thickening, neurogenesis, and inflammatory infiltrate (including neutrophils, T cells, and dendritic cells) similar to human plaque-type psoriasis (29). 

Studies have revealed that topical drug application is more effective than systemic administration for skin diseases originating under the skin. In addition, topical use also reduces systemic bioburden and, consequently, the toxic effects of drugs (30, 31). In this sense, topical treatment is the first-line approach for managing psoriasis (32). Ivermectin (IVM) is a large cyclic lactone obtained from Streptomyces composed of a mixture of 22 and 23-dihydrovermectin B1a (over 80%) and B1b (less than 20%), and (33). It is used to treat cutaneous conditions like larva migrans, scabies, filariasis, onchocerciasis, ascariasis, and strongyloidiasis (34). IVM has also been reported to restrain intense acute respiratory syndrome coronavirus 2 (SARS-CoV-2) (35). IVM can be administered locally or systemically, and its anti-inflammatory effects have been demonstrated in murine models of allergic inflammation (36, 37). The anti-inflammatory effects of IVM result from the down-regulation of kalikrein-5 (KLK5), TLR-2, cathelicidin (LL-37), and other pro-inflammatory signaling pathways (38, 39). Topical IVM has been shown to alleviate skin allergic inflammation by reducing inflammatory cytokines, activating allergen-specific T cells, and decreasing immune cell priming (37).

Carboxymethyl cellulose (CMC) is a semi-natural linear polymer, soluble in water and one of the derivatives of cellulose. In the pharmaceutical industry, it is used as a lubricant, viscosity modifier, emulsifier, and stabilizer to develop various pharmaceutical dosage formulations (40, 41). Its swelling and water absorption capacity is excellent. Being non-toxic, CMC is compatible with the skin, mucous membranes, and bones (42-44). CMC has shown significant potential in skincare products, both as a bioactive ingredient and structural component (45). Additionally, CMC is used as a template for skin regeneration and wound healing applications (42). CMC gels have been considered for developing safe drug delivery carriers in clinical fields due to their low immunogenicity, good biocompatibility, and biodegradability (46).

The present study was conducted to prepare a CMC-based gel and investigate the therapeutic effects of topical IVM on imiquimod-induced psoriasis. This research involved determining PASI (Psoriasis Area Severity Index), spleen-to-body weight index, TLR4 and NF-κB expression using immunohistochemistry, and histopathological evaluation on mice. 

## Materials and Methods

### Chemicals

Ivermectin (IVM) and carboxy methyl cellulose (CMC) were obtained from Sigma Aldrich. Aldara (comprising 5% IMQ, obtained from Meda [Solna, Sweden]). Monoclonal antibodies of mouse TLR4 ((25) sc-293072) and NF-kappa p65 (ab-16502) were used for immunofluorescence assays.

### Animals

The present study examined 15 female Wistar rats weighing 160–180 g. Mice were purchased from Elm Bavaran Aftab Company. The maintenance conditions were the same for all mice: 12 hr of light, 12 hr of darkness, and a temperature of 25 °C. All experiments in this research were conducted in accordance with the Ethics Committee of Kermanshah Faculty of Medical Sciences (Ethical code: IR.KUMS.AEC.1402.037) (47). 

### Preparation of topical gel containing ivermectin (1%w/w)

Gels are extensively applied in topical formulations for therapeutic and cosmetic objectives (48). A 2% carboxymethyl cellulose (CMC) solution in water was prepared. The solution was thoroughly mixed using a magnetic stirrer at ambient temperature for three hours. Then, IVM 1% w/w was added to the solution and was stirred (550 rpm) for an adequate time. This gel was prepared freshly and used immediately.

### Induction of imiquimod-psoriasis and ivermectin treatment

One day prior to treatment, the back skin of each mouse was shaved using hair removal creams. Then, the mice were randomly divided into three different groups (n = 5/group): 1) control group: received a standard diet every day for 7 days; 2) IMQ group: IMQ cream (1 mg per mouse) was applied evenly every day for 7 days; 3) topical IVM-gel group: on the shaved area, IVM-gel (1%) was used for 7 days. In the IVM group, IVM treatment was used on the shaved area 20 min before IMQ cream from day three to day seven of the study. The dose of IVM has been chosen based on a previous study (49). All animals were euthanized on the last day using IP injection of 10 mg/kg xylazine and 50 mg/kg ketamine, and skin samples and spleen were collected for further analysis.

### Evaluation of the score of psoriasis area severity index (PASI)

To determine the Psoriasis Area Severity Index (PASI), rats were examined over the entire 7-day period. The clinical scoring system of PASI was applied to determine the disease progression, the status of skin inflammation, its severity, and the therapeutic effects of IVM-gel (7, 41). Three parameters of erythema, silver scale (desquamation), and thickness on the skin dorsal region of all rats were examined. A score between 0 and 4 was considered for each of these parameters. 0: no sign; 1: mild; 2: moderate; 3: severe; 4: very severe. 

### Spleen to body weight index

On the last day of the study, after the PASI score was assessed, the animals’ weights were recorded. Then, after the animals were sacrificed, the spleens were carefully dissected, cleaned, and weighed. The proportion of spleen weight to body weight (organ index) was reported in g/g (50).

### Hematoxylin and eosin (H&E) staining

For histological studies, skin sections from the dorsal region were fixed with 10% formalin and dehydrated with a graded alcohol series. The samples were then embedded in melted paraffin, and 5-micron slices were made using a microtome. The samples were placed on a slide and stained with hematoxylin-eosin (E&H). Finally, stained samples were imaged at 40x magnification with a camera (50).

### TLR4 and p65 NF-κB immunostaining

Formalin-preserved skin samples were used to evaluate the expression changes of TLR4 and NF-κB through immunostaining. Five micrometer slices were placed on slides coated with saline. Then, they were deparaffinized and hydrated with ethanol (graded series) and were washed with distilled water. The slides were incubated at room temperature (2 hr) and at 2–8 °C (24 hr) with normal donkey serum (10%) containing rabbit monoclonal NFkB antibody (phospho S40, 1:100 dilution, Abcam, USA) or rabbit polyclonal TLR4 alpha antibody (Ser32/S36 phosphor, 1:100 dilution, Elabscience, USA). The slides, after washing with PBS (4 times), were incubated with goat anti-rabbit IgG (H+L) (FITC) antibody (orb688925) at a dilution of 1:150 for 1.5 hr at 37 °C. In the next step, after washing the samples with PBS and DAPI (Sigma-D9542) (3 times, 20 min) and pouring glycerol/PBS solution, they were assessed with a fluorescence microscope (Olympus) and ImageJ software was used to compute the expression of TLR4 and NF-κB (51).

### Statistical analysis 

Statistical analysis was performed using Prism 8 software. The results are reported as mean ± SEM. One-way ANOVA and post hoc statistical tests were used to compare the mean difference of different groups, and in each case *P*<0.05 was considered statistically significant.

## Results

### Effect of IVM on IMQ-induced psoriatic-like skin inflammation, alterations of bodyweight, and spleen weight

**Figure 1:**
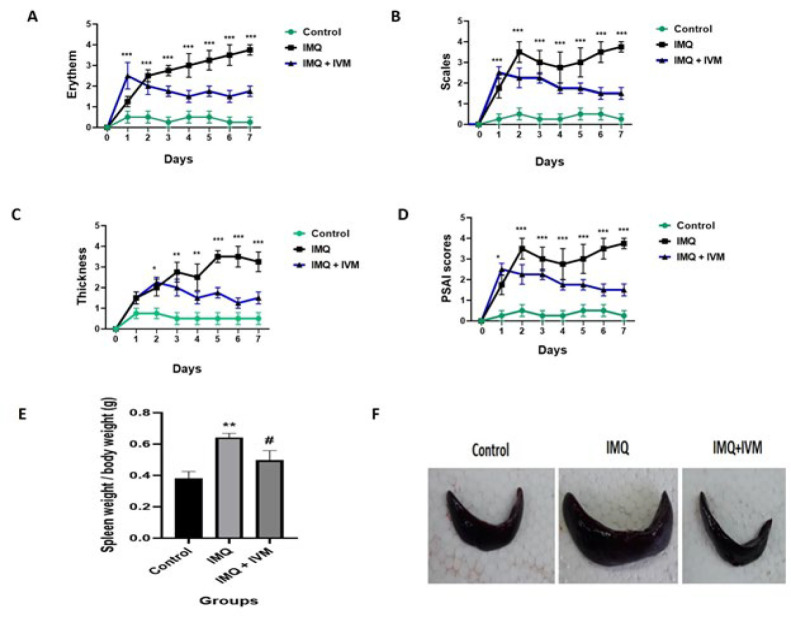
Results of the effect of topical IVM-gel in rats (1% w/w)

To assess the therapeutic effect of IVM-gel on psoriasis caused by IMQ, the changes in clinical phenotypic characteristics, including scaling, erythema, thickness, spleen/body weight, and the morphology of spleens, were evaluated (Figure 1A-F). IMQ significantly increased these parameters compared to the control. Conversely, treatment with topical IVM-gel (1% w/w) for seven consecutive days dramatically decreased the thickness, scaling, erythema, and spleen/body weight compared with the IMQ group. 

### Effect of topical IVM on TLR4 and p65 NF-KB protein expression 

**Figure 2:**
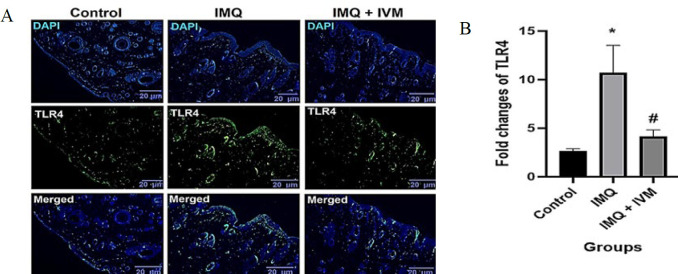
(A) TLR4 fluorescence immunostaining of the skin (× 40). (B) the optical intensity of IF assay results using ImageJ

**Figure 3:**
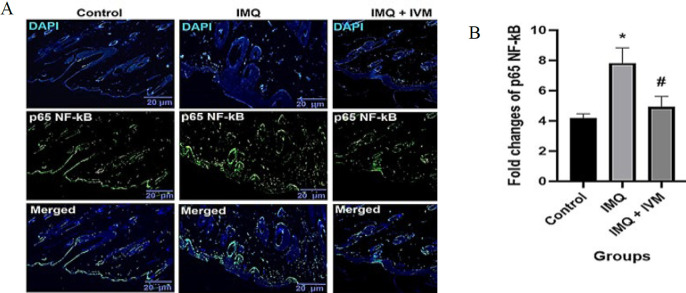
(A) p65 NF-κB fluorescence immunohistochemistry staining (IF) (× 40). (B) the optical intensity of IF assay results using ImageJ

Fluorescence immunostaining of TLR4 and NF-κB proteins to assess the effects of IMQ and IVM-gel are shown in Figures 2 and 3. In the IMQ group, the expression level of TLR4 was significantly higher than in the control group (*P*<0.05). Topical administration of IVM-gel (1% W/W) significantly ameliorated the elevated TLR4 levels observed in the IMQ group (*P*<0.05). In addition, after 7 days of IMQ administration, NF-κB protein levels were dramatically increased compared to the control group (*P*<0.05). Conversely, treatment with IVM-gel (1% W/W) significantly reduced the increased levels of NF-κB compared to the IMQ group (*P*<0.05).

### Histological results

**Figure 4:**
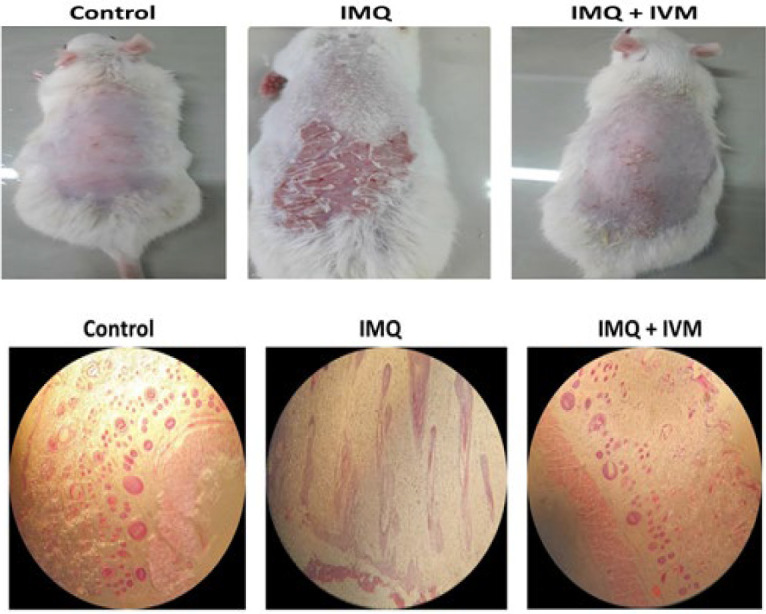
(A) Phenotype appearance of mice from control, IMQ, and IMQ-IVM groups

In the control group, the skin tissue and epiderm thickness were normal. After treatment with IMQ, the thickness of the epidermis, dermal fibrosis, and infiltration of inflammatory cells were increased. All these negative changes were reduced after treatment with IVM-gel (Figure 4 A-B).

## Discussion

 Psoriasis is a common chronic skin disease that leads to immune dysfunction in keratinocytes, resulting in delayed differentiation, rapid cell proliferation, and apoptosis (52). Branisteanu and colleagues reported that in psoriasis, there is excessive production of important inflammatory mediators such as TNFα in the skin, leading to rapid cell growth and skin damage. Blocking the production of TNF α helps to halt the inflammatory cycles of psoriasis (53). It has been found that topically applied IMQ induces psoriasis. IMQ is known as an immune response modulator that acts as a TLR-7 agonist (54). Erythema, scaling, and skin thickness are the important features of IMQ-induced psoriasis (55). In the epidermis of a mouse model caused by IMQ or psoriasis patients, the expression of cytokines such as TNF-α, IL-23, IL-17, and IL-22 is very high (55-57). The activation and increase of autoreactive skin T cells and keratinocytes through stimulation of the up-regulation of these cytokines result in a pro-inflammatory state (58, 59). In our study, topical IMQ application for seven days increased the PASI score, body weight changes, spleen weight, erythema, scaling, and skin thickness, consistent with previous studies (7, 60). In contrast, local IVM-gel (1%w/w) applied on the skin lesions of rats exhibited therapeutic effects in their skin lesions. IVM by restraining phosphorylation of the mitogen-activated protein kinases (MAPK) p38, c-Jun N-terminal kinase (JNK), and extracellular-signal-regulated kinase (ERK) 1/2 leads to the suppression of production of prostaglandin E2 (PGE2), and nitric oxide (NO) (inflammatory mediators), as well as a reduction in the expression levels of cyclooxygenase-2 (COX2) and inducible NO synthase (iNOS) (61). Recently, in phase III clinical trials, the potential of IVM for topical treatment of skin inflammatory diseases has been demonstrated. IVM cream showed strong efficacy in decreasing skin inflammatory lesions in patients with moderate to intense papulopustular rosacea (62). 

IVM has been shown to reduce inflammation by inhibiting the NF-κB pathway (63). The NF-κB signaling pathway is involved in the regulation of various cellular processes, such as inflammation and proliferation (20). NF-κB activates cytokines involved in the inflammatory response, including IL-1β, IFN-γ, TNF-α, IL-4, and IL-22 (64). The role of TLR4 in defense against microbes on the skin surface and in the pathogenesis, occurrence, and progression of psoriasis has been demonstrated in several studies (21, 53, 65). In the present study, local IVM treatment resulted in reduced inflammation and skin lesions through the down-regulation of NF-κB and TLR4 expression. TLR4 is an innate immune system receptor expressed in keratinocytes and human skin (38). The activation of TLR4 leads to downstream factor NF-κB activation and its translocation to the nucleus, resulting in increased expression of pro-inflammatory cytokines, IL-1β, TNF-α, and IL-6 (64, 66). Thus, inhibiting inflammatory responses is effective in reducing the progression of psoriasis (67). In IMQ-induced psoriasis models in mice, IMQ acts as a Toll agonist, activating Toll receptors in the body, resulting in the inflammatory response, which in turn creates psoriasis-like lesions (68). TLRs are involved in regulating NF-κB (as a downstream signaling pathway) in the immune response (69). The outcomes of Lv et al. showed that Yangxue Jiedu soup (YJS) is capable of inhibiting the NF-κB signaling pathway, blocking TLR4 activation, and restraining the secretion of HSP70 exosome, further reducing the production of inflammatory cytokines, and subsequently, contributing to the improvement of psoriasis (68). 

Fluorescence immunostaining results indicated that IMQ significantly increased TLR4 and p65 NF-κB protein expression in psoriasis model mice. In contrast, topical administration of IVM reduced inflammation by decreasing the levels of TLR4 and p65 NF-κB. Moreover, the results of H&E staining showed that IVM-gel decreased dermal fibrosis, thickness of epiderm, and infiltration of inflammatory cells caused by IMQ in animals, suggesting a therapeutic effect of IVM-gel for treating psoriasis skin lesions. 

(A) erythema, (B) scales, (C) thickness, (D) PASI score, (E) spleen/body weight, and (F) morphology of spleens of each group following IMQ-induced psoriasis in rats

*P*<0.1 vs control group;* P*<0.05 vs IMQ group. Data are presented as mean ± SD. IVM: Ivermectin; PASI: Psoriasis area severity index; IMQ: Imiquimod

*P<0.05 vs the control group; #*P*<0.05 vs the IMQ group. TLR4: Toll-like receptor 4; IMQ: Imiquimod

*P<0.05 vs the control group; #*P*<0.05 vs the IMQ group. p65 NF-κB: p65 nuclear factor kappa-B; IMQ: Imiquimod

The photograph is taken on the last day of the study. (B) Histological assay of hematoxylin and eosin staining (×40) of back skin of mice in the control, IMQ, and IMQ + IVM groups. IMQ: Imiquimod; IVM: Ivermectin

## Conclusion

Psoriasis is a common chronic and recurrent inflammatory skin disease related to the immune system, which is characterized by accelerated growth of skin cells. Topical use of IMQ is one of the common methods of inducing psoriasis in animal models, resulting in skin changes that resemble psoriatic lesions. In the present study, topical application of IVM-gel improved rats’ skin thickness, erythema, and silver scale. Moreover, IVM was able to moderate the changes in spleen weight to body weight, which is one of the side effects of psoriasis. It should be noted that the therapeutic activity of IVM is mainly due to its anti-inflammatory capacity mediated by down-regulation of the NFKB pathway, inhibiting the activation of TLR4. Based on these findings, topical IVM-gel can be used as one of the treatment options to reduce psoriasis lesions and their complications. 

## Declaration

We have not used AI tools or any technologies to prepare this manuscript.
